# Supplementary material for: A Tunable, Simplified Model for Biological Latch Mediated Spring Actuated Systems
Source: Integr Org Biol. 2022 Jul 30;4(1):obac032. doi: 10.1093/iob/obac032 (PMC9434652; doi:10.1093/iob/obac032)
Supplement: obac032_Supplemental_Files [file obac032_supplemental_files.zip › model-paper.zip!/model-paper/TITLE-PAGE-IOB-2022-015.R1.docx]

**A Tunable, Simplified Model for Biological Latch Mediated Spring Actuated Systems**

Andrés Cook^1^, Kaanthi Pandhigunta^1^, Mason A. Acevedo^1^, Adam Walker^1^, Rosalie L. Didcock^1^, Jackson T. Castro^1^, Declan O’Neill^1^, Raghav Acharya^2^, M. Saad Bhamla^2^, Philip S. L. Anderson^3^ and Mark Ilton^1∗^

^1^Department of Physics, Harvey Mudd College, Claremont, CA 91711, USA

^2^School of Chemical and Biomolecular Engineering, Georgia Institute of Technology, Atlanta, Georgia 30318, USA

^3^Department of Evolution, Ecology, and Behavior, University of Illinois at Urbana-Champaign, Urbana, IL 61801, USA

^∗^Corresponding author. Mark Ilton (milton@hmc.edu)

**Running Title:** Template Model for Biological Spring Actuated Systems

**Keywords:** biomechanics, biological springs, computational modeling
